# Morphological and dimensional analysis of the sella turcica across skeletal patterns: a cross-sectional study in an Iranian population

**DOI:** 10.1007/s44445-025-00058-3

**Published:** 2025-09-26

**Authors:** Peyman Zamanipour, Ali Bagherpour, Maryam Omidkhoda, Bizhan Shayanfar, Maryam Valizadeh, Kimia Jafarpour

**Affiliations:** 1 Department of Orthodontics, School of Dentistry, Mashhad University of Medical Sciences, Mashhad, Iran; 2 Department of Oral Radiology, School of Dentistry, Mashhad University of Medical Sciences, Mashhad, Iran; 3 Dental Research Center, School of Dentistry, Mashhad University of Medical Sciences, Mashhad, Iran

**Keywords:** Anatomy and histology, Cephalometry, Hypothalamus, Malocclusion, Sella turcica

## Abstract

That houses the pituitary gland, across various skeletal patterns in an Iranian cohort, highlighting its diagnostic potential in orthodontics and forensic identification. We examined 233 cephalometric radiographs from individuals aged 18–70 years in Mashhad, Iran (78 males (33.5%) and 155 females (66.5%)). The dimensions of the sella turcica (diameter, length, and depth) were measured using Romexis software. The Kruskal-Wallis test was used to assess dimensional differences across skeletal patterns (*p* < 0.05). Pearson’s correlation was used to analyze the relationships between dimensions and age, while the Mann‒Whitney test was used to compare dimensions between sexes. Data were analyzed using SPSS version 20 and ANOVA; normality was assessed with the Shapiro–Wilk test, and results are reported as mean ± standard deviation. Significant correlations were found between the dimensions and shape of the sella turcica. The longest length (6.78 mm) was associated with oval shapes (*P =* 0.003), whereas the greatest diameter (9.25 mm) was associated with flat shapes (*P =* 0.013). The length and diameter increased with age (*P =* 0.001 and *P =* 0.035, respectively). No significant relationships were observed with morphology, sex, skeletal pattern, or facial height (*P >*0.05). Sella turcica dimensions are influenced by age and floor shape but not by sex, morphology, or skeletal pattern. These findings provide valuable insights for cephalometric analysis to diagnose hypophyseal and craniofacial syndromes.

## Introduction

The sella turcica, which is easily identifiable on lateral cephalometric radiographs, is commonly traced via cephalometric analysis because of its significant diagnostic relevance in hypophyseal pathologies and craniofacial syndromes (Sathyanarayana et al. 2013; Antonarakis et al. 2022). Structurally, the sella turcica, which resembles a Turkish saddle, is composed of dorsum sellae, tuberculum sellae, and fossa hypophysis. It encases the pituitary gland within the hypophyseal fossa, bounded anteriorly by the tuberculum sellae and posteriorly by the dorsum sellae (Nerurkar et al. 2022). Adjacent anatomical structures include the anterior and posterior clinoid processes, which are extensions of the sphenoid bone (Sethi et al. 2023).

The sella turcica floor has been classified as round, flat, or oval, with round and oval shapes being the most prevalent (Sathyanarayana et al. 2013). Morphological variations, including sella turcica bridging and irregularities of the posterior dorsum sellae, are observed in individuals with craniofacial anomalies (Sathyanarayana et al. 2013). The literature indicates that the sella turcica typically has a length ranging from 4 to 12 mm and a diameter between 5 and 16 mm (Andredaki et al. 2007). Additionally, in an Iranian cohort, the mean sella turcica length was 9.04 mm, with an average depth of 8.03 mm (Valizadeh et al. 2015).

Variations in the size and shape of the sella turcica may indicate underlying pathological conditions. Enlargement is commonly associated with intrasellar adenomas, empty sella syndrome, or aneurysms, whereas a reduced size may suggest hypopituitarism or genetic conditions (Roomaney and Chetty 2020). Morphological anomalies such as an oblique anterior wall or pyramidal dorsum sella are more commonly observed in severe craniofacial disorders that require orthodontic or surgical intervention (Zawiślak et al. 2023).

Given the considerable variability in the dimensions of the sella turcica across populations, this study aimed to evaluate its morphology and dimensions in an Iranian cohort with varying skeletal patterns. This analysis will enhance our understanding of its diagnostic importance, particularly in the fields of orthodontics and craniofacial studies.

## Materials and methods

In this descriptive-analytical study, samples were selected from the lateral cephalograms of patients who visited the Dental School of the Mashhad University of Medical Sciences and a private radiology center in Mashhad, Iran. The study included cephalograms of 233 patients aged 18–70 years who were taken between 2020 and 2023 and who had no growth or skeletal abnormalities. Patients with skeletal anomalies, cleft lip/palate, or other craniofacial abnormalities, including bridging and solid sella turcica morphologies (excluded due to measurement challenges), were not included.

Sella turcica morphology: The morphology of the sella turcica can be classified into six groups: normal, oblique, double contour, bridging, notching, and pyramidal (Axelsson et al. 2004).

**Fig. 1:**
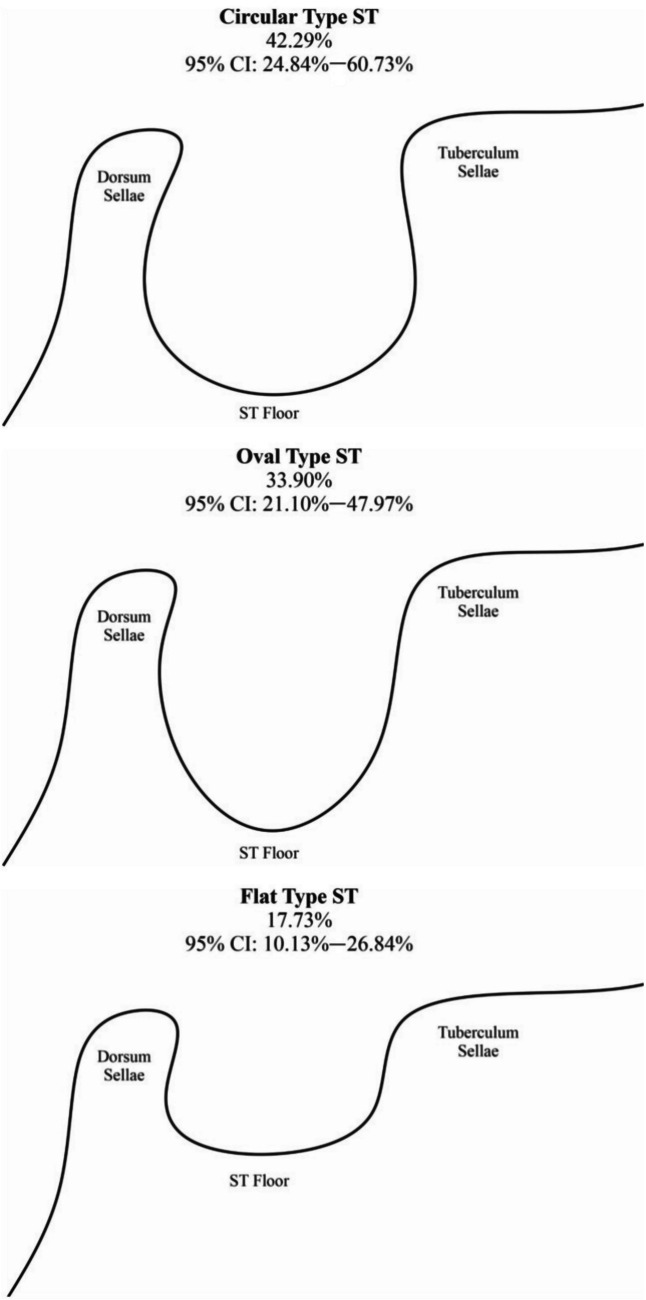
Illustration showing the oval, round, and flat types of the sella turcica floor (Iskra et al. 2023). Morphology of the sella turcica: a meta-analysis based on the results of 18,364 patients. Brain Sciences;13(8):1208) (Reproduced from Iskra et al. (2023) with permission from Brain Sciences)

Additionally, as shown in Fig. 1, the shape of the sella turcica floor was categorized as oval, round, or flat in the midsagittal view.

In this study, we distinguish “shape” as the two-dimensional contour of the sellar floor (classified as round, flat, or oval), whereas “morphology” refers to the overall anatomical form of the sella turcica, including aspects such as bridging, notching, and irregularities of the posterior dorsum sellae.

Skeletal patterns (Classes I, II, and III) were determined based on the ANB angle. The typical ANB angle for a Class I skeletal pattern is around 2 degrees (ANB 0°–4°). An angle exceeding 4 degrees points to a Class II skeletal pattern, whereas an angle below 0 degrees indicates a Class III skeletal pattern.

Skeletal patterns were analyzed via AudaxCeph software (ver. 6.1.4), which also measures the SNA, SNB, ANB, and SN-FH angles. In cases where landmarks were unclear or the SN-FH angle was abnormal (more or less than 6°), consultation with an orthodontist was conducted, and adjustments were made if necessary.

The vertical angle was also analyzed via the FMA (Frankfort Mandibular Plane Angle) from the Tweed analysis model. An FMA angle between 23° and 27° was considered normal facial height; values above this range indicated long facial height, whereas values below this range were classified as short facial height.

### Imaging and assessment

**Fig. 2:**
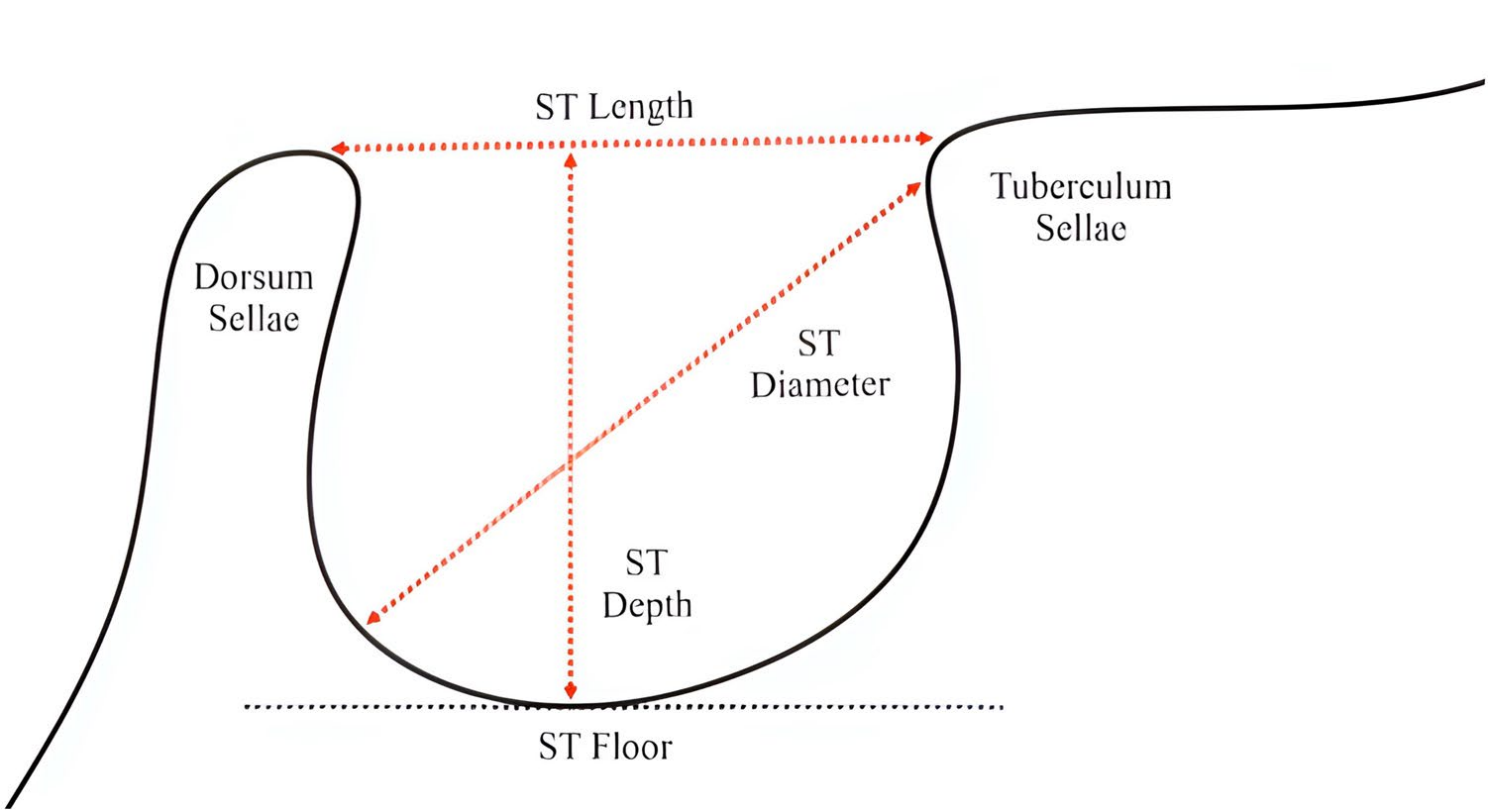
Illustration showing the dimensions of the sella turcica (Iskra et al. (2023) Morphology of the sella turcica: a meta-analysis based on the results of 18,364 patients. Brain Sciences.;13(8):1208) (Reproduced from Iskra et al. (2023) with permission from Brain Sciences)

All images were obtained via a Vatech device (PCH-2500, Vatech, South Korea) and EzDent-i software. The photos were reviewed by an oral and maxillofacial radiology specialist and a student (BS) in a semidark room via a 19-inch Philips LCD monitor. Inter-rater reliability was evaluated using the kappa coefficient and demonstrated a high level of agreement (κ = 0.90, *p* < 0.05). Similarly, intra-rater reliability was assessed and showed strong consistency (κ = 0.87, *p* < 0.05). The data were subsequently subjected to further analysis. Sella turcica Dimensions in sagittal sections of the selected lateral cephalograms and the length, diameter, and depth of the sella turcica were measured via Romexis software (Planmeca, Finland). Length was defined as the distance between the dorsum sellae and tuberculum sellae. The diameter was measured as the distance from the tuberculum sellae to the posterior-most point of the posterior wall of the hypophysis. Depth was measured as the perpendicular distance from the line connecting the dorsum and tuberculum sellae to the deepest point of the sellar floor (Fig.2).

### Sample size estimation and statistical analysis

The sample size was determined based on the study by Afzal et al. (aine Shabbir et al. 2021) using G*Power software (ver. 3.1.9.7), assuming an effect size of 0.3 for comparisons among three skeletal pattern groups (Class I, II, and III). A minimum of 40 samples per group was required to ensure adequate statistical power.

Data were analyzed using SPSS version 20. Normality was assessed via the Shapiro‒Wilk test. The results are reported as means ± standard deviations. The Kruskal‒Wallis test was used to compare the sella turcica dimensions across skeletal patterns (p<0.05). Pearson correlation and Mann‒Whitney tests were used to analyze the relationships between the sella turcica dimensions and age and sex, respectively.

### Ethical considerations

No additional exposure was administered to the patients in this study, and radiographic findings were reported confidentially in aggregate form. The study was conducted under Research Project No. 4012062 (approved on April 12, 2023) with ethics approval No. IR.MUMS.DENTISTRY.REC.1401.159 (granted on March 14, 2023).

## Results

In the present study, 233 lateral cephalograms were obtained. Table 1 summarizes the frequency and distribution of sex, skeletal classification (based on ANB angle), facial height, sella turcica morphology, and shape. Among the participants, 155 were female (66.5%). Class II skeletal pattern (ANB > 4°) was the most frequent, occurring in 38.2% of the cases. Long facial height was the most prevalent feature, observed in 39.1% of cases. The most common sella turcica morphology was normal (59.7%), followed by double contour (18.9%). The predominant shape of the sella turcica was round, with a frequency of 57.1%.

**Table 1:** Analysis of baseline information among participants

Variations		Number (%)
Sex	Male	78(33.5)
	Female	155(66.5)
Skeletal Classification	I	65(27.9)
	II	89(38.2)
	III	79(33.9)
Facial Height	Normal	67(28.8)
	Long	91(39.1)
	Short	75(32.2)
Morphology	Normal	139(59.7)
	Oblique	30(12.9)
	Double Contour	44(18.9)
	Notching	14(6)
	Pyramidal	6(2.6)
Shape	Oval	5(2.1)
	Round	133(57.1)
	Flat	95(40.8)

As shown in Table 2, the sella turcica was longer in males (6.09 mm) than in females (5.87 mm), but this difference was not statistically significant (*P =* 0.722). The diameter was 9.01 mm in males and 8.66 mm in females, with no significant difference (*P =* 0.365). The depth of the sella turcica was 7.98 mm in men and 7.78 mm in women, with no statistically significant difference between the sexes (*P =* 0.283). The Shapiro–Wilk test confirmed that the distributions of the sella turcica dimensions were normal (*p >* 0.05 for all).

**Table 2:** Analysis of Sella Turcica Dimensions (Length, Diameter, and Depth) by sex, skeletal classification (by ANB angle), facial height, morphology, and shape

		Length of sella turcica						Diameter of sella turcica						Depth of sella turcica					
		Mean	SD	median	Max	Min	Result of the statistical test	Mean	SD	median	Max	Min	Result of the statistical test	Mean	SD	median	Max	Min	Result of the statistical test
Sex	Male	6.09	2.72	6.00	14.20	1.60	0.722^a^	9.01	2.26	9.00	14.00	4.10	0.365^a^	7.98	1.49	7.90	12.70	5.00	0.283^a^
	Female	5.87	2.50	5.40	13.00	1.00		8.66	2.05	8.30	13.50	1.50		7.78	1.29	7.80	11.40	4.90	
Skeletal Classification	I	6.21	2.68	5.70	13.00	1.60	0.158^b^	9.19	2.13	9.00	13.50	5.30	0.111^b^	7.89	1.46	7.90	11.40	4.90	0.898^b^
	II	5.53	2.51	5.20	13.50	1.00		8.46	2.01	8.20	12.90	1.50		7.79	1.23	7.70	11.00	5.00	
	III	6.18	2.53	6.00	14.20	1.00		8.79	2.21	8.40	14.00	4.10		7.87	1.43	7.90	12.70	5.10	
Facial Height	Normal	6.17	2.76	6.10	12.70	1.50	0.595^c^	9.11	2.40	9.00	14.00	5.00	0.524^c^	7.81	2.28	8.00	10.90	5.20	0.962^c^
	Long	5.92	2.60	5.30	14.20	1.00		8.60	2.03	8.30	13.20	4.10		7.88	1.44	7.70	11.40	4.90	
	Short	5.75	2.38	5.30	12.40	1.20		8.69	1.97	8.40	12.90	1.50		7.48	1.36	7.90	12.70	5.00	
Morphology	Normal	6.02	2.59	5.70	13.50	1.20	0.679^a^	8.79	2.17	8.60	14.00	1.50	0.088^a^	8.00	1.24	8.00	11.40	5.10	0.776^a^
	Oblique	5.88	2.30	5.60	10.20	1.00		9.19	1.95	9.30	12.40	4.50		7.35	1.25	7.25	12.10	5.00	
	Double Contour	5.98	2.32	5.80	12.70	1.50		8.57	1.95	8.05	13.50	5.00		7.86	1.56	7.70	12.70	5.20	
	Notching	4.96	2.59	5.00	9.90	1.60		8.57	2.25	7.95	12.80	5.60		7.72	1.73	7.60	10.90	4.90	
	Pyramidal	6.35	5.00	4.95	14.20	1.00		8.46	3.26	8.10	12.60	4.10		7.03	1.38	7.15	8.80	5.40	
Shape	Oval	6.78	4.23	8.90	10.60	1.00	0.003^b^	6.68	3.77	7.30	11.10	1.50	0.013^b^	7.84	1.86	7.50	10.00	5.20	0.67^b^
	Round	5.51	2.56	5.20	14.20	1.00		8.52	2.01	8.20	14.00	4.10		7.78	1.41	7.70	12.70	4.90	
	Flat	6.50	2.40	6.40	13.00	1.70		9.25	2.07	9.30	13.50	4.80		7.94	1.27	7.90	11.20	5.00	

^a^: results of the Mann-Whitney U test

^b^: results of ANOVA

^c^: results of the Kruskal-Wallis test

Class I skeletal pattern (ANB 0°–4°) exhibited the longest, widest, and deepest sella turcica dimensions*,* with average values of 6.21 mm, 9.19 mm, and 7.89 mm, respectively. However, these differences were not statistically significant (length, *P =* 0.158; diameter, *P =* 0.111; depth, *P =* 0.898).

Among the facial height categories, individuals with normal facial height generally exhibited the largest sella turcica dimensions, except for depth, where those with long facial height showed the greatest measurement, with mean values of 6.17 mm for its longest dimension and 9.11 mm for its diameter. However, these differences were not statistically significant (length, *P =* 0.595; diameter, *P =* 0.524; depth, *P =* 0.962).

In terms of morphology, the longest sella turcica was observed in individuals with pyramidal morphology, whereas the widest was observed in those with oblique morphology. However, no significant relationship was detected between the morphology and dimensions of the sella turcica (length *P =* 0.679, diameter *P =* 0.088, depth *P =* 0.776).

For the sella turcica shape, the longest sella turcica was associated with an oval shape, whereas the shortest sella turcica shape was associated with a round shape. Significant correlations were found between the shape of the sella turcica and its length and diameter (*P =* 0.003 and *P =* 0.013, respectively).

As shown in Table 3, a direct correlation was found between age and the dimensions of the sella turcica. Length and diameter were significantly correlated with age (*P =* 0.001 and *P =* 0.035, respectively), whereas depth was not (*P =* 0.393).

**Table 3:** Analysis of the correlation between age and sella turcica dimensions

	Length Of Sella Turcica	Diameter Of Sella Turcica	Depth Of Sella Turcica
Pearson correlation coefficient	0.210	0.136	0.056
*P* Value	0.001	0.035	0.393

## Discussion

Our study provides important insights into the dimensions and morphology of the sella turcica, revealing notable correlations with variables such as age and shape. As age increased, we observed significant changes in the length and diameter of the sella turcica. Interestingly, factors traditionally considered in orthodontic assessments—such as sex, vertical facial height, and skeletal pattern—did not show significant influence on these dimensions.

The sella turcica is a saddle-shaped depression in the sphenoid bone that houses the pituitary gland. It forms early during skull development and reflects broader patterns of craniofacial growth. During embryogenesis, the sella region plays a critical role as a conduit for neural crest cell migration into the frontonasal and maxillary fields. These neural crest cells contribute to both the formation of the sella turcica and the development of dental and maxillary structures (Sathyanarayana et al. 2013). Disruptions in this migration process can result in lasting craniofacial anomalies (Zawiślak et al. 2023). Since the morphology of the sella turcica becomes largely fixed by puberty, congenital variations—such as dorsum sellae bridging or irregular sella walls—tend to persist into adulthood (Bavbek and Dincer 2014). These structural anomalies often coincide with other developmental defects; for example, irregularities in the anterior sella wall have been associated with midface anomalies, while posterior wall notching may suggest intracranial abnormalities (Kjær et al. 1999). Given that the cranial base, including the sphenoid bone, derives from both neural crest and paraxial mesodermal origins, the sella turcica’s variability in size and shape reflects its complex embryological development (Zawiślak et al. 2023).

The hypothalamus and pituitary gland begin forming prenatally from two distinct embryonic tissues (Musumeci et al. 2015). The anterior hypothalamus originates from neural crest cells, while the posterior portion arises from paraxial mesoderm. Development begins around the seventh week of gestation, and anomalies arising during this period often persist throughout life. Therefore, precise measurement of these structures’ dimensions and shapes is essential in diagnosing various pituitary disorders (Kjær et al. 1999).

Lateral cephalometric radiography, primarily used for skeletal/dental assessment, can also detect craniofacial anomalies in typically young, healthy orthodontic patients (Patel 2022). Notably, with 13% of brain tumors occurring in the hypothalamic region, precise examination of craniofacial structures, including the hypothalamus, during these radiographs becomes crucial. Understanding the diverse shapes of the hypothalamus is vital for distinguishing between normal developmental variations and potential pathological conditions (Luzzi et al. 2023).

Our findings align with prior research indicating that the dimensions of the sella turcica, including length, diameter, and depth, do not differ significantly by sex. This consistency is observed across various populations, such as the Norwegian, Saudi Arabian, and Turkish populations. However, a Turkish study has reported minor diameter differences between the sexes (Issrani et al. 2023; Magat and Sener 2018; Axelsson et al. 2004).

Age, on the other hand, showed a significant positive correlation with sella turcica dimensions in our study, corroborating findings from Bosnian, Iraqi, and Saudi Arabian populations (Alkofide 2007; Muhammed et al. 2019; Yassir et al. 2010). This trend suggests that age-related changes in the dimensions of the sella turcica are a common physiological phenomenon, highlighting the need for further research into specific age-related changes.

In contrast, we found no significant relationships between sella turcica dimensions and malocclusion type, vertical growth pattern, or skeletal classification. Although these results align with previous studies that also reported no substantial connections between these variables but it might be because of our small sample size (Alkofide 2007; Khojastepour et al. 2012; Valizadeh et al. 2015). Some studies—particularly those focusing on Class III malocclusions—have documented minor dimensional differences (Motwani et al. 2017). For instance, Class II malocclusions typically show smaller anteroposterior dimensions and depth compared to Class III.

Additionally, we did not identify significant relationships between the morphology and dimensions of the sella turcica. While varying prevalence of morphologies such as pyramidal or bridging shapes has been reported, no consistent link with sella turcica dimensions has been established. However, in another Iranian cephalometric study, pronounced morphological differences were observed (Valizadeh et al. 2015).

Additionally, we did not identify significant relationships between the morphology and dimensions of the sella turcica. While varying prevalences of morphologies such as pyramidal or bridging shapes have been reported, no consistent link with sella turcica dimensions has been established. This suggests that although morphological variations are common, they do not significantly influence the overall dimensions of sella turcica across different populations (Andredaki et al. 2007; Valizadeh et al. 2015; Alkofide 2007).

### Diagnostic and clinical implications

 Because the sella turcica houses the pituitary, its evaluation serves dual roles. Radiographically, the sella point (“S”) is a fundamental cephalometric landmark. Sella turcica can be precisely traced on lateral skull radiographs, enabling accurate measurement of its length, depth, and diameter (Nabavizadeh et al. 2023). Since the sella turcica matures before the mandible, its dimensions can help predict future jaw growth: for example, a relatively shallow or constricted sella in a young patient might foreshadow Class II (retrognathic mandible) tendencies, whereas a larger sella could indicate Class III (prognathic mandible) patterns (Tepedino et al. 2019).

Importantly, persistent sella anomalies often accompany systemic conditions. Many pituitary or skeletal syndromes manifest with characteristic sella changes. For instance, large-scale surveys show that about two-thirds of healthy individuals have a “normal” sella, but the remaining third exhibit variations (bridging, notching, etc.) that can signal underlying malocclusion or pathology. In orthodontic patients, identifying an abnormal sella early can prompt evaluation of endocrine or craniofacial disorders and influence treatment planning (Nabavizadeh et al. 2023). Consequently, detailed cephalometric assessment of the sella turcica – recording its shape and precise dimensions – is essential. Such measurements not only refine the diagnosis of skeletal malocclusion patterns but also ensure that any clinically significant deviations (even those asymptomatic) are detected for timely intervention (Konwar et al. 2016).

A major strength of this study is the dual analysis of size and shape in an underrepresented Iranian population, adding valuable normative data. However, we acknowledge several limitations. First, the two-dimensional nature of lateral cephalograms restricts full visualization of the sella’s three-dimensional morphology. Second, some subgroups—particularly oval shape and pyramidal morphology —had relatively small sample sizes, potentially limiting statistical power in subgroup comparisons. Third, although shape classifications are derived from previously validated studies, the categorization of sella turcica morphology and floor shape involves a degree of subjectivity. Despite using established criteria, interobserver variation is possible and may affect consistency in shape classification.

Our findings reinforce that chronological age and prenatal morphogenesis dictate sella turcica dimensions, while sex, malocclusion, vertical facial height, and skeletal pattern do not. Clinically, precise cephalometric analysis of the sella turcica—readily obtained on routine orthodontic radiographs—offers both a stable landmark for growth assessment and an early warning for potential pituitary or craniofacial pathology. By integrating sella measurements into diagnostic workflows, clinicians can enhance the accuracy of treatment planning, particularly in borderline or complex malocclusion cases where subtle base‑of‑skull variations may influence mandibular growth predictions. Looking forward, advanced imaging modalities (CBCT, MRI) and quantitative shape‑analysis tools will enable volumetric assessment of the sella turcica, reduce subjectivity in morphology classification, and further elucidate links between pituitary anatomy and craniofacial development.

## Data Availability

The datasets used and analysed during the current study are available from the corresponding author on reasonable request.
